# Plasma pharmacokinetics after combined therapy of gemcitabine and oral S-1 for unresectable pancreatic cancer

**DOI:** 10.1186/1756-9966-29-15

**Published:** 2010-02-24

**Authors:** Bunzo Nakata, Ryosuke Amano, Shigetomi Nakao, Tatsuro Tamura, Osamu Shinto, Toshiki Hirakawa, Yoshihiro Okita, Nobuya Yamada, Kosei Hirakawa

**Affiliations:** 1 Department of Surgical Oncology, Osaka City University Graduate School of Medicine, Osaka 545-8585, Japan

## Abstract

**Background:**

The combination of gemcitabine (GEM) and S-1, an oral 5-fluorouracil (5-FU) derivative, has been shown to be a promising regimen for patients with unresectable pancreatic cancer.

**Methods:**

Six patients with advanced pancreatic cancer were enrolled in this pharmacokinetics (PK) study. These patients were treated by oral administration of S-1 30 mg/m^2 ^twice daily for 28 consecutive days, followed by a 14-day rest period and intravenous administration of GEM 800 mg/m^2 ^on days 1, 15 and 29 of each course. The PK parameters of GEM and/or 5-FU after GEM single-administration, S-1 single-administration, and co-administration of GEM with pre-administration of S-1 at 2-h intervals were analyzed.

**Results:**

The maximum concentration (Cmax), the area under the curve from the drug administration to the infinite time (AUCinf), and the elimination half-life (T1/2) of GEM were not significantly different between GEM administration with and without S-1. The Cmax, AUCinf, T1/2, and the time required to reach Cmax (Tmax) were not significantly different between S-1 administration with and without GEM.

**Conclusion:**

There were no interactions between GEM and S-1 regarding plasma PK of GEM and 5-FU.

## Background

Unresectable pancreatic cancer is known to have a poor prognosis, with most patients dying within several months of diagnosis. However, recent progress in chemotherapy using gemcitabine (GEM) for this disease has improved patient survival. A number of phase III clinical trials have been performed to determine the GEM regimens that lead to the greatest increases in survival compared with GEM monotherapy. To date, only one regimen has been shown to yield significantly longer survival periods than GEM alone in phase III studies: GEM with erlotinib, an epidermal growth factor receptor (EGFR)-targeting agent [1].

S-1 is an oral fluoropyrimidine derivative that contains tegafur (a 5-FU prodrug) and a reversible competitive dihydropyrimidine dehydrogenase (DPD) inhibitor, 5-chloro-2,4-dihydrogenase (CDHP). As DPD is a rate-limiting enzyme that degrades 5-FU, CDHP is expected to enhance the cytotoxicity of 5-FU by prolonging high 5-FU concentrations in blood and tumor tissues [2]. In Japan, S-1 has been clinically used as a first-line chemotherapeutic agent for pancreatic cancer since being approved for national health insurance coverage in 2006. A phase II study of S-1 for 40 patients with metastatic pancreatic cancers resulted in the response rate of 37.5% and the overall survival time of 9.2 months [3].

As the efficacy of S-1 monotherapy against pancreatic cancer is not satisfactory, numerous studies using S-1 combined with GEM have been conducted. Two phase I studies and two phase II studies of the combination therapy showed promising efficacy and acceptable adverse events [4-7]. A phase III study comparing GEM+S-1 vs. S-1 monotherapy vs. GEM monotherapy for metastatic pancreatic cancer (GEST study) has been underway in Japan and Taiwan since 2007. In contrast to the large number of clinical trials regarding GEM+S-1, pharmacokinetic studies to investigate the interaction between the two agents have been very limited. This is the first study to compare the plasma pharmacokinetics (PK) of GEM and 5-FU after GEM+S-1 to those after single administration of individual drugs in the same patients.

## Methods

### Eligibility

Patients under 80 years of age with a diagnosis of unresectable pancreatic cancer were eligible. Eastern Cooperative Oncology Group performance status (PS) ≤ 2, and life expectancy ≥ 12 weeks were required. Patients were required to have measurable or assessable disease and to have had no chemotherapy or immunotherapy before enrolling. Other eligibility requirements included adequate bone marrow function (Hb ≥ 9.0 g/dl, white blood cells between 4,000 and 12,000/μl, neutrophils ≥ 2,000/μl and platelets ≥ 100,000/μl), total bilirubin ≤ 2 mg/dl, AST and ALT ≤ 100 IU/l, alkali phosphatase ≤ 2 times the upper normal level, and BUN and serum creatinine ≤ the upper normal level.

### Patients

A total of six patients with unresectable pancreatic cancer diagnosed by imaging studies including abdominal dynamic computed tomography were enrolled in this study between April and June, 2007. Mean age ± standard deviation was 68 ± 4 years (range, 63-73 years). One case had liver metastasis, three had peritoneal metastasis, and two had tumors involving the celiac and/or superior mesenteric arteries. Informed consent from all participants was obtained. The institutional review board for human experimentation in our hospital approved the study protocols.

### Treatment

S-1 (Taiho Pharmaceutical Co., Tokyo, Japan) was administered orally at a dose of 30 mg/m^2 ^twice daily after a meal. One course consisted of consecutive administration for 28 days, followed by a 14-day rest period. GEM 800 mg/m^2 ^in 100 ml normal saline was administered intravenously (i.v.) for 30 min on days 1, 15 and 29 of each course. The regimen was set by referring to previous clinical trials [4-7].

### Sample collection

**Figure 1:**
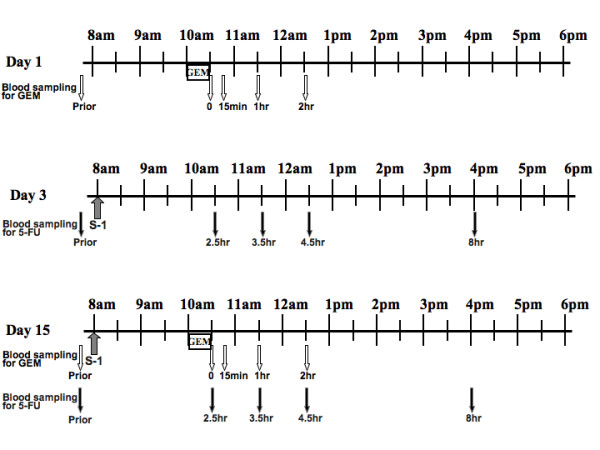
**Drug administration and blood sampling schedule**.

Blood samples were drawn on days 1, 3 and 15 of the first course. The object of sampling at day 1 was to monitor the plasma PK of GEM after administration of GEM alone. Subsequently, S-1 administration on day 1 of the first course began at the evening after blood samplings. The object of sampling at day 3 was to monitor the plasma PK of 5-FU after administration of S-1 alone. The object of sampling at day 15 was to examine the changes in individual drug PK after other drug administration. For this purpose, S-1 was administered 2 h before administration of GEM (Figure 1), when the plasma concentration of 5-FU had increased substantially [8]. Each peripheral blood sample (2 ml) was collected into a heparinized tube that contained 20 μl of tetrahydrouridine (a cytidine deaminase competitive inhibitor) solution at a concentration of 10 mg/ml. The samples were centrifuged at 3000 rpm for 10 min. Plasma was stored at -20°C until the measurement of 5-FU and GEM concentrations.

### GEM assay

The high-performance liquid chromatography (HPLC) system consisted of a Waters 2690 liquid chromatograph separation module and a Waters SMH column heater (all from Waters (MA, USA). The Atlantis^R ^dC18 column (150 × 4.6 mm; particle size, 5 μm; Waters) was used for the peak separation of GEM. The HPLC mobile phase was a solution of 5 mM phosphate buffer (pH 7.2). The ultraviolet detector was a Waters 2487 (Waters), and was used at 272 nm. Plasma samples were deproteinized with 20% TCA, and the supernatants were filtered using Ultrafree-MC (Nihon Millipore, Tokyo, Japan) with pore diameters of 0.20 μm. Aliquots of 20 μl were injected into the HPLC system. The quantitative range of this method was 50-40000 ng/ml.

### 5-FU assay

The high-performance liquid chromatographic-mass spectrometry (LC/MS) system consisted of a Micromass ZQ-2000 mass spectrometer, a Waters 2695 liquid chromatograph separation module and a Waters SMH column heater (all from Waters). The Atlantis^R ^dC18 column (150 × 2.1 mm; particle size, 5 μm; Waters) was used for the peak separation of 5-FU. The HPLC mobile phase was a solution mixed purified water and acetonitrile. The mass spectrometer was used in the negative ESI mode. The detector was used in SIR mode with a selected ion recording procedure at m/z = 128.9 for 5-FU and at m/z = 130.9 for 5-FU-^15^N_2_. To plasma samples, internal standard solution (including 5-FU-^15^N_2_) was added, and was then extracted with ethyl acetate. The organic layer was evaporated to dryness under a stream of nitrogen. The residue was dissolved in purified water, and after vortex mixing, the mixture was filtered using Ultrafree-MC (Nihon Millipore) with pore diameters of 0.20 μm. Aliquots of 20 μl were injected into the LC/MS system. The quantitative range of this method was 5-500 ng/ml.

### Statistical analysis

The area under the curve from the drug (S-1 or GEM) administration to the infinite time (AUCinf) was calculated according to the trapezoidal rule using the WinNonlin program (Ver. 5.2; Pharsight Co., Mountain View, CA, USA). Two-sided paired Student's *t*-test on log-transformed plasma concentration data was used to compare the maximum concentration (Cmax) and AUCinf between single administration and co-administration. The two-sided paired Student's *t*-test was conducted on the elimination half-life (T 1/2) and time required to reach Cmax (T max) in order to compare data for single administration and co-administration. A *P *value of < 0.05 was considered to be statistically significant.

## Results

### Clinical outcome

Five of six patients were treated by GEM+S-1 for 5 to 16 courses (median, 8 courses). However, it was necessary to reduce the doses of S-1 and/or GEM by approximately 25% due to grade 3 or more neutropenia for two patients after one course, and for two patients after three courses. The regimen was stopped at the end of one course in one patient who could not continue oral intake of S-1 due to developing the stenosis at Treitz ligament by cancer invasion. The MST of total patients studied was 12.5 months, ranging from 3 to 22 months. The 1-year survival rates were 67%. One partial response was observed. SPan-1, one of reliable tumor marker for pancreatic cancer [9], titers in sera were decreased 50% or more in all of 5 patients who had abnormal level of SPan-1 prior to the treatment.

### Plasma PK

**Figure 2:**
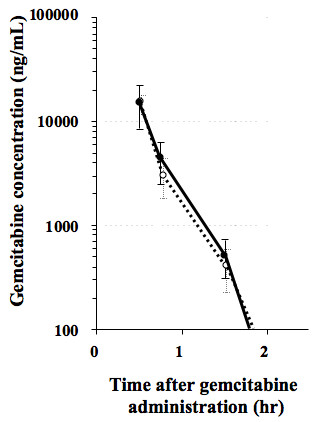
**Plasma concentrations of gemcitabine (GEM) after administration of GEM 800 mg/m^2 ^alone (open circles) and GEM 800 mg/m^2 ^+ S-1 30 mg/m^2 ^(closed circles)**.

**Figure 3:**
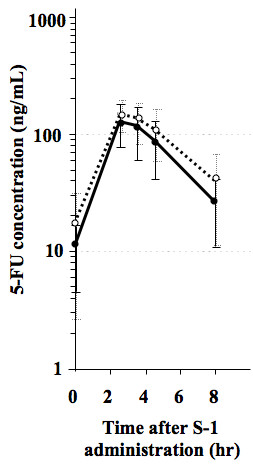
**Plasma concentrations of 5-fluorouracil after administration of S-1 30 mg/m^2 ^alone (open circles) and GEM 800 mg/m^2 ^+ S-1 30 mg/m^2 ^(closed circles)**.

There were no significant differences between plasma PK parameters of GEM after administration of GEM alone and GEM+S-1 (Table 1, Figure 2). There were no significant differences between the plasma PK parameters of 5-FU after administration of S-1 alone and GEM+S-1 (Table 2, Figure 3).

**Table 1:** Comparison of pharmacokinetic parameters of gemcitabine (GEM) in plasma between administraion of GEM alone and GEM+S1

	Cmax (ng/ml)	AUCinf (hXng/ml)	T1/2 (h)
Day 1	15833 ± 2477	8467 ± 1092	0.12 ± 0.033
(GEM alone)			
Day 15	14924 ± 5828	8384 ± 2915	0.153 ± 0.069
(GEM+S-1)			
P-value	0.604	0.7406	0.1594

GEM was intravenously given at a dose of 800 mg/m2. S-1 was orally given at a dose of 30 mg/m2.

Cmax, maximum plasma concentration;

AUCinf, area under plasma concentration-time curve from time zero to infnite time; T1/2, elimination half-life.

Titers are expressed as means ± SD (n = 6). P-values were examined by two-sided paired t-test after log-transformation.

**Table 2:** Comparison of pharmacokinetic parameters of 5-fluorouracil in plasma between administration of S-1 alone and gemcitabine (GEM)+S1

	Cmax (ng/ml)	AUCinf (hXng/ml)	T1/2 (h)	Tmax (h)
Day 3	162 ± 46	853 ± 329	1.96 ± 0.73	3.16 ± 0.81
(S-1 alone)				
Day 15	135 ± 56	682 ± 256	2.22 ± 0.84	3.07 ± 0.53
(GEM+S-1)				
P-value	0.8644	0.2063	0.604	0.1683

GEM was intravenously given at a dose of 800 mg/m2 S-1 was orally given at a dose of 30 mg/m2.

Cmax, maximum plasma concentration;

AUCinf, area under plasma concentration-time curve from time zero to infnite time; T1/2, elimination half-life; Tmax, the time required to reach Cmax.

Titers were expressed as means ± SD (n = 6). P-values were examined by two-sided paired t-test after log-transformation.

## Discussion

For the last decade, GEM monotherapy has been the standard chemotherapy regimen to treat advanced pancreatic cancer. The drug has an approximately 5% response rate and improves MST to less than 6 months [10]. Clinical trial data has demonstrated a response rate of 44-48% and an MST of 10.1-12.5 months when S-1 is administrated with GEM [6,7]. The efficacy of this combination therapy, including our regimen, thus appears to be better than GEM monotherapy, although a true evaluation requires data from the ongoing phase III trial (GEST study).

Our results demonstrated that pre-administration of S-1 did not increase Cmax, AUCinf or T1/2 of plasma GEM (Table 1, Figure 2). Nakamura *et al*. performed a PK study of GEM with S-1; S-1 was given orally at a dose of 30 mg/m^2 ^twice daily for 14 consecutive days, followed by a 1-week rest. GEM 1000 mg/m^2 ^was given in a 30-min i.v. on day 8 and day 15. In six patients with metastatic pancreatic cancer, the PK parameters of Cmax and AUCinf for GEM were examined on day 8. It was concluded that their data were similar to those of GEM single-administration, as determined in a phase I study [11] carried out by other investigators [12].

The sample size affects the statistical accuracy, however, the ethical matters limit the sample size. There have been some reports statistically comparing the PK parameters between two groups composed of five or six patients [13,14]. In our study on six patients, the statistical analysis was done to detect the relative change of the PK parameters in individual patients using the paired Student's *t*-test. In this analysis, the statistical power depends on the intra-individual variance and not on the inter-individual variance.

Correale *et al*. reported that pre-administration of GEM had an effect on the plasma PK of 5-FU [15]. In their study, 20 patients with metastatic gastroenteric carcinomas were treated with 30 min i.v. of 5-FU 400 mg/m^2 ^and folinic acid (FA) 100 mg/m^2 ^at 1 h after 30 min i.v. of GEM 1000 mg/m^2^. The control group (5-FU/FA group) consisted of 16 patients with gastroenteric carcinomas receiving 30 min i.v. of 5-FU 400 mg/m^2 ^and FA 100 mg/m^2^. The AUC of plasma 5-FU in GEM+5-FU/FA group was approximately twice as high as that in 5-FU/FA group. The Cmax and T1/2 of 5-FU in GEM+5-FU/FA group were higher than those in 5-FU/FA group. The enhanced 5-FU systemic exposure in the presence of GEM may induce severe adverse events as well as high levels of antitumor activity. In fact, a clinical phase I/II trial testing GEM+5-FU/FA for 51 patients with gastroenteric cancers reported frequent grade 4 gastroenteric toxicity and two treatment-related deaths [15].

In contrast to the study by Correale *et al*., in our examination, the plasma Cmax, AUCinf and T1/2 of 5-FU after co-administration of S-1 with GEM showed no increases when compared to those after S-1 single-administration (Table 2, Figure 3). Although significant differences were not shown, the mean values of Cmax and AUCinf of 5-FU at day 15 were lower than those at day 3 (Table 2). The reason is obscure, however, continuous administration of S-1 might affect 5-FU pharmacokinetics.

In the catabolic pathways, 5-FU is degraded by DPD. As S-1 contains a very strong DPD inhibitor, CDHP, the Cmax and AUCinf of plasma 5-FU after S-1 administration may reach the limit expected by the amount of tegafur present in S-1. The affects of GEM metabolites on Cmax and AUC of plasma 5-FU after S-1 administration may be little lower than expected based on the presence of CDHP in plasma. The above-mentioned mechanism may explain our results that PK parameters of plasma 5-FU after S-1 administration did not differ with and without GEM administration. Moreover, no enhancement of 5-FU systemic exposure after S-1 administration in the presence of GEM may be an advantage in reducing the frequency of adverse events [16].

The synergistic effects of S-1 and GEM may be explained by the following mechanism occurring in tumor cells. S-1 is converted into 5-FU. An active metabolite of 5-FU is fluorodeoxyuridine monophosphate (FdUMP), which inhibits DNA synthesis by forming of ternary complex with 5,10-methylene tetrahydrofolate and thymidylate synthase. GEM inhibits ribonucleotid reductase, a key enzyme in the salvage pathway of pyrimidine biosynthesis. Consequently, GEM reduces the synthesis of deoxyuridine monophosphate, a major competitor of FdUMP, resulting enhancement of 5-FU cytotoxicity [17]. Another potential mechanism is that 5-FU leads to an increase in cell surface human equilibrative nucleoside transporter 1 (hENT1) [18,19]. The most active GEM uptake is via hENT1. Thus, increased hENT1 expression by 5-FU may augment GEM cytotoxicity by increasing GEM concentrations in tumor cells.

In conclusion, the present study obtained by the limited number of patients demonstrated the combination chemotherapy of S-1 with GEM did not affect the PK of each drug. As S-1 combined with GEM may be a promising regimen, further investigations should be carried out to elucidate the synergistic mechanisms between the two drugs.

## List of abbreviations

GEM: gemcitabine; 5-FU: 5-fluorouracil; PK: pharmacokinetics; Cmax: maximum concentration; AUCinf: area under the curve from the drug administration to the infinite time; T1/2: elimination half-life; Tmax: time required to reach Cmax; EGFR: epidermal growth factor receptor; DPD: dihydropyrimidine dehydrogenase; CDHP: 5-chloro-2,4-dihydrogenase; MST: median survival time; PS: performance status; i.v.: intravenously; HPLC: high-performance liquid chromatography; LC/MS: high-performance liquid chromatographic-mass spectrometry; FdUMP: fluorodeoxyuridine monophosphate; hENT1: human equilibrative nucleoside transporter 1.

## Competing interests

The authors declare that they have no competing interests.

## Authors' contributions

BN have made substantially contribution to conception, design, data analysis, interpretation of data, and drafting the manuscript. RA, SN, TT, OS, TH, and YO have made substantial contributions to patients sample collection and acquisition of data. NY and KH have made contributions to revising the manuscript critically for important intellectual content. All authors read and approved the final manuscript.
